# Extracellular Vesicle Protein Expression in Doped Bioactive Glasses: Further Insights Applying Anomaly Detection

**DOI:** 10.3390/ijms25063560

**Published:** 2024-03-21

**Authors:** Mauro Nascimben, Hugo Abreu, Marcello Manfredi, Giuseppe Cappellano, Annalisa Chiocchetti, Lia Rimondini

**Affiliations:** 1 Center for Translational Research on Autoimmune and Allergic Diseases, Department of Health Sciences, Università del Piemonte Orientale UPO, 28100 Novara, Italy; mauro.nascimben@uniupo.it (M.N.); hugo.abreu@uniupo.it (H.A.); giuseppe.cappellano@med.uniupo.it (G.C.); annalisa.chiocchetti@med.uniupo.it (A.C.); 2 Interdisciplinary Research Center of Autoimmune Diseases, Department of Health Sciences, Università del Piemonte Orientale UPO, 28100 Novara, Italy; 3 Biological Mass Spectrometry Laboratory, Department of Translational Medicine, Università del Piemonte Orientale UPO, 28100 Novara, Italy; marcello.manfredi@uniupo.it

**Keywords:** proteomics, distance function, mass spectrometry, isolation forest, anomaly detection

## Abstract

Proteomic analysis of extracellular vesicles presents several challenges due to the unique nature of these small membrane-bound structures. Alternative analyses could reveal outcomes hidden from standard statistics to explore and develop potential new biological hypotheses that may have been overlooked during the initial evaluation of the data. An analysis sequence focusing on deviating protein expressions from donors’ primary cells was performed, leveraging machine-learning techniques to analyze small datasets, and it has been applied to evaluate extracellular vesicles’ protein content gathered from mesenchymal stem cells cultured on bioactive glass discs doped or not with metal ions. The goal was to provide additional opportunities for detecting details between experimental conditions that are not entirely revealed with classic statistical inference, offering further insights regarding the experimental design and assisting the researchers in interpreting the outcomes. The methodology extracted a set of EV-related proteins whose differences between conditions could be partially explainable with statistics, suggesting the presence of other factors involved in the bioactive glasses’ interactions with tissues. Outlier identification of extracellular vesicles’ protein expression levels related to biomaterial preparation was instrumental in improving the interpretation of the experimental outcomes.

## 1. Introduction

Mass spectrometry (MS, Appendix B.1) is an analytical technique used in proteomics to identify and quantify proteins within a biological sample. It generates complex data, and bioinformatics tools are essential for processing, analyzing, and interpreting this information. Integrating both numeric outcomes and advanced computational methods is crucial for extracting meaningful insights from MS data; for example, anomaly detection techniques have implications for identifying proteins with outlier levels or detecting defective proteins [1,2]. For anomaly detection, software programs like EnsMOD (https://github.com/niaid/EnsMOD, accessed on 21 December 2023) can be used to analyze any omics dataset with normally distributed variance, including proteomics datasets [3]; it can remove abnormal protein levels before running statistics on the data to purge outliers. AnomalP is an anomaly detection approach for detecting anomalous protein folding, which is crucial for determining defective and dysfunctional proteins [4]; this program leverages deep autoencoders for reconstructing information about the structural similarity. Additionally, anomaly detection in proteomics datasets has been studied in the context of mixed high-dimensional molecular data, where most anomaly detection algorithms identify complete samples as outliers or anomalies [5]. Furthermore, robust subspace methods have been developed for outlier detection in genomic data, which can also be applied to proteomics datasets [6]. Overall, anomaly detection in proteomics is a complex and essential area of research that has implications for understanding protein structure, protein–protein interactions, and identifying defective proteins; the literature encloses benchmarks and ad hoc analysis sequences for this task [7,8,9]. However, identifying anomalies of protein expression levels within small-dimensional datasets presents additional difficulties because the dataset is more susceptible to variability and noise, making it challenging to distinguish between actual biological variations and random fluctuations; anomalies may be masked by inherent variability [10]. Adopting specific solutions to match the characteristics of the data under exam could ensure the reliability of anomaly detection in small datasets; indeed, small datasets may not adequately represent the diverse biological conditions, making it challenging to distinguish between normal variation and true anomalies. Also, traditional statistical methods may be less robust when dealing with small datasets hiding outliers that may not reach significance [11].

### 1.1. Notes on Small Sample Inference

In general, during proteins’ expression levels analysis, statistics based on *p*-values are commonly used to assess significant differences in their expression levels between experimental conditions. Through statistics, the goal is to demonstrate whether any observed difference is statistically significant or could have occurred due to random chance. The *p*-value is the probability of observing a test statistic as extreme as, or more extreme than, the one calculated from the actual data, assuming the hypothesis of no real difference between the groups being compared [12]. In other words, it informs how likely the observed differences in protein expression could have occurred due to random chance alone [13]. In the case of small *p*-values (typically below a predetermined significance level, often denoted as α , such as 0.05), statistics suggest that the observed differences are unlikely to be due to chance and are more likely due to a natural effect. A small *p*-value does not prove a significant difference between the groups being compared; it simply indicates that the observed data are unlikely under the assumption of the null hypothesis (no difference in protein expression between the Control and Treatment groups) [14]. Additionally, *p*-values are subject to factors such as sample size and experimental design [15,16,17], which prejudice their interpretation, and, in general, proteomics studies often involve relatively small sample sizes compared to genomics studies. The choice of small samples could be due to several factors: large samples can be resource-intensive in terms of time, cost, and data processing, manage the biological variability, or experimental design of proteomics studies, which may focus on the in-depth characterization of a few samples. However, running statistics on small samples might be sub-optimal [18,19,20]. Findings may not generalize well to the larger population, leading to imprecise results: attempting complex analyses with insufficient data may drive unreliable conclusions [21]. Small sample sizes often result in low statistical power, which is the probability of detecting a true effect when it exists. Low power increases the likelihood of Type II errors, where one may fail to detect a real effect even if it is present. Also, the probability of making a Type I error (rejecting a true null hypothesis) is inflated with small samples: small datasets are more likely to produce spuriously significant results due to random variability [22]. With small samples, individual data points can significantly impact the overall results: the increased variability can generate less-stable estimates of parameters and unreliable statistical inferences, not generalizing well to the larger populations [23]. The risk of obtaining results specific to particular individuals or conditions when working with a small sample is higher, and these findings may not be applicable to a broader context. Additionally, small samples are more sensitive to the influence of outliers or extreme values: a single outlier can disproportionately affect the results. Small sample sizes make detecting small or subtle effects challenging: even if a true effect exists, it may be difficult to distinguish from random variability when dealing with a small dataset. For all these reasons, describing the results obtained on a small sample might be better than extracting a general rule through statistics applicable to a population, and machine-learning methods might offer instruments suitable for this aim. Outliers, or anomalies, are data points that deviate significantly from most of the data, and several machine-learning techniques and algorithms have been specifically designed for this purpose [24,25,26]. The possibility of detecting proteins whose expression levels show diverging behavior between experimental conditions, even in a small dataset, might support and offer further insights into the laboratory outcomes. The example in Appendix A.1 highlights the following aspect: while it is technically possible to conduct a *t*-test with a small sample size (e.g., less than five), there are several important considerations and potential issues associated with doing so [27]. The *t*-test assumes that the data are approximately normally distributed, and with very small sample sizes, the distribution of the data becomes critical. It should be considered how small sample sizes reduce the statistical power of a study: the power of a statistical test is the probability of detecting a true effect if it exists. With low power, there is a higher chance of making a Type II error (failing to reject a false null hypothesis) because Type II errors are inversely related to statistical power. Additionally, while a *t*-test may be more robust to violations of normality with larger sample sizes, a small sample size necessitates a very large effect size for the test to have reasonable power [28]. Another aspect is the generalizability of the results. Findings from studies working on few samples are often less generalizable to the broader population: it is essential to consider whether the results can be extrapolated beyond the specific sample studied [29]. For this reason, considering supplementary methods to accompany standard statistical approaches may be appropriate for small samples. A last remark pertains to the specific concepts of statistical significance and biological (or clinical) significance [30]. Statistical significance measures whether the results observed in a study are likely to be due to a natural effect or if they could have occurred by chance employing a threshold. Achieving statistical significance does not necessarily mean that the observed effect is practically or clinically significant; it only indicates that the observed differences are unlikely to be due to random chance. Clinical significance, on the other hand, is concerned with the practical importance or relevance of the study findings in real-world terms. It focuses on whether the observed effects are large enough to be meaningful or impactful in a clinical or practical sense. Even if a study produces statistically significant results, the effect size (magnitude of the difference) could be considered to determine if it has clinical relevance.

### 1.2. Observations on Distance Functions

Distance functions can be a valuable complementary approach to statistical significance in specific contexts, especially when dealing with non-parametric or distribution-free methods. Distance-based measures focus on quantifying the similarity or dissimilarity between data points rather than relying on assumptions about the underlying data distribution. Distance functions often operate at the level of individual data points, and in some cases, statistical significance testing and distance functions can complement each other. The detection of data points with extreme behavior could be simplified through distance functions. These points, which could be labeled as rare or anomalies (also called outliers, Appendix B.2), can stem from many factors, ranging from natural fluctuations in the data and inaccuracies in the measurement process to infrequent and unusual occurrences during the data collection phase. Indeed, outliers can manifest in various ways, and different types of outliers are conceivable based on their characteristics and impact on the data. Euclidean distance is sensitive to the numeric values of protein abundances: being the absolute differences between expression values also measured by *t*-test statistics, it could be the natural choice to select for connecting the results to t-statistics. Among the various functions applicable to independent experiments, Euclidean distance proved practical in reducing false positives compared to the rank product method in small samples [31]. Also, in a series of works [32,33,34,35], the association between Euclidean distances and Spearman correlations proved to be effective in building clusters for anomaly detection in proteomics data. When studying intensities profiles of MS spectra for shape similarity search, cosine distance might be a common choice [36]. Cosine distance measures the cosine of the angle between two vectors, focusing on the direction rather than the magnitude of the vectors. It is robust to differences in overall magnitude and is suitable when measuring directional changes in protein expression. Another advantage of cosine distance is the applicability to sparse data. Instead, Euclidean distance considers both the direction and magnitude of the vectors; thus, the focus is on magnitude rather than direction. In the proposed investigation, the Euclidean distance sensitivity to the numeric values of protein abundances looks suited to spotting differences between experiments. The application of the Euclidean distance over the example in Appendix A.1 has been demonstrated in Appendix A.2, showing proteins that have close distances despite one of them not being statistically significant between theoretical experiments.

### 1.3. Remark for Statistical Analysis on Primary Cell Data

Primary cells, directly isolated from living organisms or tissues, tend to be less stable than established cell lines. The reasons for this are the limited proliferative capacity (primary cells have a finite lifespan and can only divide a certain number of times before they undergo senescence or cell death), heterogeneity, increased sensitivity to variations in culture conditions compared to cell lines, genetic instability, and different response to experimental treatments or conditions [37]. Compared to cell lines, primary cells closely resemble in vivo conditions and are often used to study specific biological processes, conduct drug testing, or investigate disease mechanisms. Statistical analysis of primary cell data presents challenges compared to cell lines: the analysis of primary cell data may need to account for and manage their inherent variability [38]. Obtaining primary cells usually involves working with a limited number of samples, especially when dealing with human or animal tissues; indeed, small sample sizes can affect the statistical power of experiments and increase the risk of obtaining results that may only be representative of some of the population. Also, if primary cells are collected from different donors, there can be significant donor-to-donor variation regarding genetic background, health status, and other factors. Another difficulty pertains to temporal variability: primary cells might change their behavior over time in culture, reflecting temporal dynamics that can complicate statistical analyses. Finally, primary cells often have limited proliferative capacity, which can restrict the number of replicates available for experiments. This limitation can impact the reliability of statistical analyses, particularly for complex experimental designs or when investigating subtle effects [39].

### 1.4. Aim of the Study

Proper identification of outliers allows the development of novel biological hypotheses that are not considered when experimental data are initially evaluated [40]. The present research aims to test a data mining pipeline to identify proteins that exhibit abnormal behavior on biomaterial datasets with the following characteristics:

1. The procedure is studied for small-dimensional datasets
2. The procedure should support researchers in obtaining additional information on the data under analysis, marking expression levels in actual sample space rather than considering average values
3. It leverages Euclidean geometry isomorphism, taking into account the magnitude and direction of the protein level changes
4. It exploits existing and already verified methods for novel and augmented interpretation of the experimental variables
5. Verify the procedure on data coming from primary cells directly collected from donors

The aim was to exploit a specific anomaly detection analysis to evaluate abnormal extracellular vesicles’ protein content connected to the preparations of bioactive glasses. Whether protein expression levels reach statistical significance, anomaly detection offers evidence about the sample under analysis because it can measure actual donors’ values. This descriptive approach to the dataset under investigation might avoid assumptions regarding the behavior of the protein levels inside a population and report the actual findings when few samples are involved in laboratory experiments. Identified proteins might be marked as anomalies and further evaluated in light of the experimental design and research question.

The suggested computational sequence has been tested on an experimental setting involving extracellular vesicle protein expression levels obtained by culturing mesenchymal stem cells over different biomaterials. The goal is to detect a restricted set of proteins whose activation could follow over- or under-expression patterns in different donors without comparing the average levels but actual values. Moreover, this alternative approach might offer insights regarding proteins with aberrant levels in small datasets whose effect size might not be adequate for correctly interpreting some statistical outcomes.

### 1.5. Proteomics of Extracellular Vesicles

Extracellular vesicles (EVs) play a crucial role in various physiological and pathological processes. However, the protein content of these vesicles can create obstacles in their detection and analysis [41]. For instance, EVs contain a limited number of proteins compared to the parent cells: many proteins are present at low concentrations, making their detection challenging, especially when using mass spectrometry-based techniques [42]. Additionally, EVs are a heterogeneous population consisting of different subtypes such as exosomes, microvesicles, and apoptotic bodies: each subtype may have distinct protein profiles, making it challenging to obtain a comprehensive analysis [43]. Moreover, isolation methods may not separate each subtype; EVs have a broad size range (30 nm to several micrometers), and traditional proteomic techniques may not be sensitive enough to detect proteins in the lower size range. Furthermore, larger contaminants may interfere with the analysis because contamination with proteins from the culture medium or other cellular components can occur during the isolation of EVs, thus confounding the proteomic results [44]. Also, the protein composition of EVs can change dynamically in response to various stimuli or cellular conditions; this dynamic nature makes it challenging to capture a static snapshot of the EV proteome [45]. Other factors include the lack of standardized protocols (different isolation methods may yield different results impacting proteomic analysis) and post-translational modifications, affecting protein function, stability, and interactions [46].

To better understand the physiological and pathological functions of extracellular vesicles and their clinical applications, it is essential to know the cellular processes that govern their biology. The inherent properties of native/biological extracellular vesicles offer stability and enable them to cross biological barriers; this makes them an effective means of intercellular communication that can regulate function and phenotype. However, scalability and standardization of generation, molecular characterization for design and regulation, therapeutic potency assessment, and targeted delivery must be improved to realize their therapeutic potential fully. These limitations can be overcome by utilizing advanced technologies to analyze extracellular vesicles quantitatively.

### 1.6. Biomaterials Dataset Analyzed for Protein Anomaly Detection

The dataset under analysis explored the protein content of extracellular vesicles derived from mesenchymal stem/stromal cells (MSCs) from three donors cultured in contact with bioactive glasses. Mesenchymal stem cells are a type of adult stem cell found in various tissues, such as bone marrow, adipose tissue, and umbilical cord. They can uniquely differentiate into multiple cell types, including bone, cartilage, fat, and muscle cells. This differentiation potential makes MSCs promising for tissue repair, regeneration, and cell-based therapies. In addition to their differentiation capacity, MSCs exhibit immunomodulatory and anti-inflammatory properties: they can secrete various bioactive molecules that influence the immune response. Extracellular vesicles are small membrane-bound structures released by cells. They include exosomes and microvesicles, and they play a crucial role in cell-to-cell communication by transferring bioactive molecules, such as proteins, nucleic acids (RNA and DNA), and lipids, between cells [47]. EVs have gained considerable attention for their potential as therapeutic agents due to their ability to convey signaling molecules and therapeutic cargo to target cells. EVs derived from MSCs (MSC-EVs) have garnered particular interest because they can carry their parent cells’ regenerative and immunomodulatory properties. The therapeutic potential of MSC-EVs for tissue regeneration involves promoting tissue repair and regeneration by delivering growth factors and signaling molecules to damaged tissues. Furthermore, biomaterials have been extensively used to enhance tissue healing, particularly in the musculoskeletal system. Either as scaffolds or hydrogels that also act as physical support for cells or in the form of nanoparticles as delivery and protection vehicles, metallic alloys, ceramics (particularly bioactive glasses), and polymers present a promising strategy to improve treatment efficacy. For that, MSC and MSC-EVs are being increasingly studied in conjunction with different biomaterials for bone [48] and cartilage regeneration [49]. The laboratory approach consisted of culturing MSCs on top of bioactive glass discs supplemented with different metallic ions, namely silver, copper, and tellurium, which can improve the biomaterials’ osteogenic properties and accelerate the tissue regeneration process. EVs released by these preconditioned MSCs were isolated, and their protein content was investigated by mass spectrometry and then analyzed using the suggested machine-learning method.

## 2. Results

The proteins displaying Euclidean distance above the average plus one standard deviation of the whole set of proteins (empirical threshold) were considered extremely changing and collected for the Control versus Doped bioactive glasses conditions in the following tables (Table 1, Table 2 and Table 3). The tables highlight proteins with *p* < 0.05, which reject the null hypothesis, considering that the null hypothesis of the *t*-test asserts that, on average, there is no systematic difference between the two groups; conversely, any observed difference is attributed to random variation or sampling error. The tables also marked proteins whose *p*-values were between 0.05 and 0.1 as a reference because the lack of significance might be due to the small sample size, and increasing the dimension of the subjects involved might affect the statistical outcomes [50].

The most relevant protein changes discovered by the suggested procedure on Plastic or Doped bioactive glasses are shown in Table 4, Table 5 and Table 6. For example, in Table 4 and Table 5, most keywords are related to keratin, a fibrous structural protein that plays a crucial role in the regeneration and maintenance of various tissues, especially epithelial ones.

**Figure 1:**
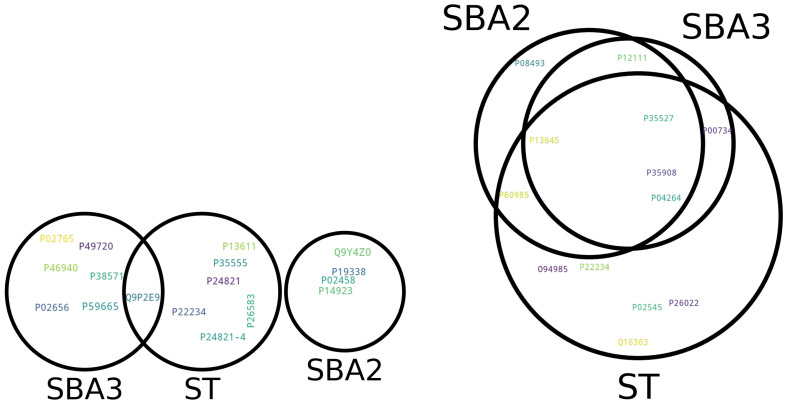
Venn diagrams of extremely changing proteins as identified by Isolation Forest and Euclidean distances. On the (**left**), the Control versus Doped bioactive glass shows only one protein in common between “SBA3” and “ST”. On the (**right**), the Plastic versus Doped conditions sharing several proteins.

Venn diagrams were created to summarize the proteins that are common to multiple experiments or those that are unique to specific conditions [51]. Venn diagrams can be used to represent the relationships between different protein sets, aiding in the identification of unique and shared proteins. In Figure 1, the two diagrams are valuable for understanding the commonalities and differences in protein expression of the identified “anomalies” across biomaterials.

The comparative analysis of the Control versus Doped condition showed only one protein whose Euclidean distance was classified as an anomaly in the presence of metal ion doping, meaning each doped biomaterial activated a peculiar set of rare proteins with specific characteristics. Instead, the Plastic versus Doped condition shared several proteins, with four highly over- or under-expressed in all laboratory preparations. The interpretation could be that shared proteins may represent core biological processes, while unique proteins may indicate context-specific EV-related biological responses.

### 2.1. Relation between Euclidean Distances and Fold Change

**Figure 2:**
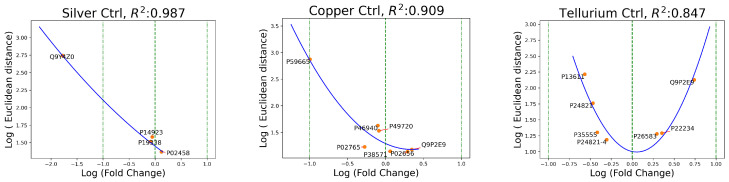
The blue line is the quadratic fit of the log fold change and Euclidean distances (ctrl versus doped distances).

In proteomics, fold change refers to the ratio of the abundance or expression levels of a particular protein in two different conditions or experimental groups. In Figure 2, the logarithmic fold change was plotted on the *x*-axis and the most evident Euclidean distances over the *y*-axis. As a reference, a green vertical line marked the value of the *x*-axis, defining no change in protein abundance between conditions, while a positive log fold change indicates an increased expression and a negative log fold change indicates a decrease. When analyzing such scatter plots, it is important to look for patterns that may reveal trends in the data. For instance, if there are proteins that show large fold changes but have low Euclidean distances, this could suggest coordinated changes in their expression. On the other hand, if there are proteins that have high Euclidean distances, it may indicate significant differences between the experimental conditions being compared. Therefore, examination of scatter plots can help identify patterns and trends in the data, which can inform further analysis and interpretation.

In Figure 2, the quadratic fit (U-shaped blue line) between the two variables was included to emphasize the presence of a nonlinear pattern in the fold change versus Euclidean metric relationship that cannot be adequately captured by a linear model (a straight line).

**Figure 3:**
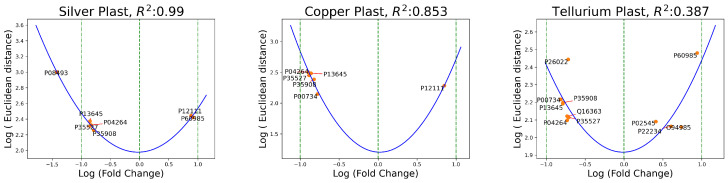
The blue line is the quadratic fit of the log fold change and Euclidean distances (plast versus doped distances).

The same type of relation was encountered in the distances between the plast and doped conditions, as shown in Figure 3.

Fold change provides information about the magnitude of differences in protein expression, and the aberrant proteins detected as anomalies match a U-shaped fit in most experiments. These graphs help define the direction of the change (increase or decrease) to aid the interpretation of the results.

### 2.2. Within-Group Variation

Within-group variation, also known as within-group variability or intra-group variation, is a measure of the dispersion or spread of individual data points within a specific experimental condition. The variance for the Control and Doped experimental sessions is shown in Table 7, with the last column reporting the absolute difference.

The SBA2 Q9Y4Z0 protein had the highest difference in variances ctrl versus doped. Other relevant changes were spotted on P59665 in SBA3 and P13611 in ST conditions. Comparing the three biomaterials, mean variances after metal doping are quite stable (Table A3). The variance for the plast and doped experimental session is shown in Table 8. In such a situation, the variance is relatively stable.

The two tables reflect a peculiar degree of variability among individual observations within each outlier protein. The difference in variance on selected proteins is more evident in the ctrl versus doped experiments (especially on Silver material). A low within-group variation suggests that the individual observations within each group are relatively similar or homogeneous; this indicates that the experimental conditions have a consistent effect. Instead, some proteins of the ctrl versus doped experiment differ greatly between experimental conditions (i.e., Q9Y4Z0, P59665, and P13611), which might have also affected *t*-test outcomes. While variance measures absolute variability, the coefficient of variation (CV) provides a measure of relative variability by normalizing the standard deviation with respect to the mean. Table A4 confirms a trend of inconstancy involving the Silver biomaterial compared to the doped one. Perhaps silver glass has a more pro-apoptotic effect and might stabilize cell proliferation, at least in the first days of culture, until the silver is almost all released from the glass surface [52]. The analysis sequence proposed in this study highlighted this aspect in the identified abnormal proteins, suggesting that the researchers should carefully evaluate the experimental outcomes considering the factors (biological and technical) that might have determined the increased variability. For example, it is worth noting that the technical variation in the process of two-dimensional electrophoresis results in a CV of 20–30% [53].

## 3. Discussion

Machine-learning anomaly detection is a technique used to identify patterns or instances that deviate from the expected behavior in a given dataset. The present study proposed a sequence of operations to perform anomaly detection on a proteomic dataset employing primary cells from three donors targeting EV protein content of experiments involving doped or not biomaterials. The objective is to detect unusual or abnormal points that could indicate potential rare occurrences: anomalies are data points that differ from most of the data, making them stand out. For anomaly detection, the Isolation Forest algorithm finds anomalies by randomly selecting a feature and then choosing a split value between the maximum and minimum values of that feature [54].

Biomaterials play a crucial role in tissue engineering because they provide a scaffold or framework for developing, repairing, or replacing tissues in the human body. Applying the suggested analysis sequence data could help researchers identify a restricted set of proteins with a peculiar role in tissue engineering by assisting in evaluating experimental outcomes. Usually, verifying if a protein’s expression is magnified or not by an experimental condition is performed through statistics or by thresholding the fold change. Although anomalies, also known as outliers, are data points that differ from most of the dataset, statistical significance testing is often used to determine whether observed effects or differences in data are likely genuine or could have occurred by chance. Technically speaking, the two approaches are different: ML-based anomaly detection often involves training models to learn the expected behavior of a system and then identifying instances that deviate from this learned normal behavior. On the contrary, statistical significance testing relies on mathematical models and statistical techniques to assess whether observed differences or effects in a sample are likely to be representative of the broader population or if they might result from random variability [55]. Also, the assumptions of the two methods are different. Anomaly detection methods typically make fewer assumptions about the underlying distribution of the data because they focus on learning patterns directly from the data and can be effective in scenarios where the distribution is complex or unknown. Statistical tests assume a specific distribution for the data, and the validity of the results can be influenced by the appropriateness of these assumptions.

The most frequent statistical outcome considered in research is the *p*-value, which is a measure that helps researchers assess the evidence against a null hypothesis. The relationship between sample size and *p*-values is crucial to statistical analysis [56,57]. As the sample size increases, statistical tests become more sensitive to detecting differences or effects. This increased sensitivity can be attributed to the reduction in sampling variability, leading to more precise estimates of population parameters. Also, effect size matters because a larger sample size could be needed to achieve statistical significance in situations where the effect size is small [58,59]. Another consideration regarding sample size involves the issue that a larger sample size might reduce the risk of Type II errors (false negatives), as the increased power improves the ability to detect true effects, but researchers should be cautious about the increased risk of Type I errors (false positives) when interpreting small *p*-values [60]. In this situation, the clinical or practical significance of the findings should be considered. Non-parametric or noncentral t-distributions could indeed be employed in case of violation of normality when dealing with few samples; however, the significance computed through a *t*-test is still commonly used because the Welch *t*-test or rank transformation before running a *t*-test might pose other challenges when dealing with small samples (reduced statistical power or increased false positives) [27]. The analysis sequence proposed in the current study could support researchers in identifying proteins that are not completely detected by statistics in situations where only a few data are available. A restricted number of samples not only affects *p*-values but also the correct identification of the confidence intervals or the reproducibility of the experimental results, which has already been observed in the literature [20,27,61,62]. However, increasing the sample size might only sometimes be possible. Working with human cells, especially primary ones, can be resource-intensive in terms of time and resources; using a smaller number of samples can help streamline experiments, making them more manageable within budgetary and time constraints [63]. With few samples, it may be easier to maintain consistent conditions across experiments and design experiments that directly test biological hypotheses without unnecessary complexity. Considering that using a limited number of samples, including three, is a common practice, new tools for analyzing and interpreting the results might help researchers support their findings. The proposed analysis pipeline evaluates donors’ cell profiles without assuming any distribution. Another aspect to consider when working on data from few donors relates to the variability in the findings: biological variability between different cultures can be substantial. One could assume that with few samples, the inherent experimental variability might be reduced, making it easier to detect experimental effects. However, this might only be true sometimes, and high variability in a few sample experiments might pose challenges and affect the convertibility of the outcomes to the human population. The current study provides further evidence on a dataset under investigation because descriptive ML-based analysis on actual data might support statistical inference or help researchers focus their attention on specific results, being more careful in interpreting the outcomes.

It could be possible that the aberrant behavior identified by the procedure might not have a solid biological explanation. However, the information provided should not be ignored because it could allow researchers to evaluate the single-subject variability of the proteins under investigation more carefully. Table 7 and Table 8 report the variance within subjects. If proteins have high within-group variation, it may indicate that individual samples within a group are responding differently, making it more challenging to attribute observed differences to specific experimental factors. Proper randomization, replication, and control of confounding variables can contribute to reducing within-group variation. The current procedure might attract the researcher’s attention to the results found in this situation, also in the light that one assumption of the *t*-test is the homogeneity of the variances [64]. Slight within-group variation is desirable, as it indicates that the observations within each group are relatively consistent. Sources of variability could be biological or technical due to laboratory procedures. Biological variability could arise from the cell cycle stage, influencing protein expression or due to genetic variations among individuals. Cell lines are generally homogeneous and genetically stable, meaning the cells in the population are very similar in terms of genetic makeup and cellular characteristics; however, in the case of primary cells, as in our study, donor-specific features, such as age, gender and effects associated with pathologies, strongly contribute to the variable protein expression commonly detected [65]. External sources of variability could be introduced by external stimuli, stressors, or environmental changes that alter cells’ protein expression profiles [66]. Regarding environmental factors, changes in the availability of nutrients and growth factors in the cell culture environment can impact protein expression. Also, variations in oxygen levels (hypoxia or normoxia) can affect cellular metabolism and protein expression, and together with other environmental conditions, such as temperature and pH, they can influence protein stability and expression [67]. Post-translational modifications are chemical modifications that occur on proteins after they are synthesized during translation: these modifications play crucial roles in regulating protein structure, function, localization, and interactions. These modifications can introduce variability: changes in the extent or pattern of modifications can influence protein function and detection. Other sources of variability in pharmacological studies are exposure to drugs or other perturbations that can lead to changes in protein expression. Technical sources of variability pertain to experimental design and execution. Variability can be introduced during sample collection, handling, and preparation; differences in sample processing techniques can lead to protein extraction and quantification variations [68]. Also, experiments conducted in different batches or on different days may exhibit batch effects, causing variations in protein profiles. Inconsistencies in technical procedures, such as variations in mass spectrometry or other analytical techniques, can contribute to variability.

As the Section 1.5 mentions, traditional proteomic techniques may not be optimized for EVs’ unique characteristics [69], and enriched analysis could support deeper biological understanding. Also, newer technologies and methodologies, such as improved mass spectrometry approaches to recognize molecular networking from MS data [70], which can reveal interactions or regulatory processes, and single-vesicle analysis techniques, are continually being developed to address these challenges [71]. Ongoing research efforts are focused on refining protocols and producing innovative techniques to improve the accuracy and reproducibility of proteomic analysis of extracellular vesicles, such as performing extensive quantitative analysis to compare populations of proteins with different features [72]. Advances in the field will likely lead to a better understanding of the role of EVs in various biological processes and their potential applications in diagnostics and therapeutics [73].

A final clarification is about the concept of anomaly, which does not imply statistical significance in protein expression. The procedure shown in the current manuscript defines proteins with abnormal behavior based on Euclidean distance metric targeting experiments involving biomaterials’ protein expression levels in primary cells. Abnormally expressed proteins (i.e., far from the normal) could be statistically significant in different experimental conditions, but it is not mandatory. As shown in Table 7 and Table 8, some identified proteins display high variance between conditions, suggesting considering this aspect when discussing the results. The goal of this methodology is to attract the attention of the researchers to specific proteins, offering further support in understanding the experimental conditions and insights into the experimental design. The supportive methodology developed in the current manuscript is not conceived as a replacement for statistics but as assistive technology to gather additional insights on a dataset under investigation.

### 3.1. Biological Interpretation of the Outcomes

When working with primary cells, it is essential to consider the common inter-donor variability often detected: it reflects the different physiological states of the subjects, which are derived from their age, gender, lifestyle, pathological condition, etc. [74]. With that, protein expression may become highly variable among donors, impairing the typical *p*-value analysis. However, an opposite response to treatment between individuals, or in this case of cell-cultured with a biomaterial, in terms of protein expression, can be pretty relevant because it can allow for the categorization of patients in groups (responders and non-responders, for example) and a better treatment selection.

In the scope of our study, comparing the bioactive glasses doped with metal ions with the respective undoped conditions, several proteins were detected as extremely changing. However, only one was in common between different conditions: Ribosome-binding protein 1 (Q9P2E9). In particular, Q9P2E9, which was altered in both copper- and tellurium-doped bioactive glass conditions, was reported to be upregulated in MSCs undergoing osteoblastic differentiation [75], which can indicate that the doping with these ions could modulate this pathway. By checking the raw data and considering the classical analysis based on *p*-value, we verified that MSC culture in contact with CuSBA3 and STe5 leads to an overexpression of Q9P2E9 encapsulated in EVs, therefore indicating a possible osteogenic-promoting effect of the ion doping.

Despite no other proteins in common between conditions, comparing the STe5 with its undoped counterpart revealed three other proteins simultaneously differentially expressed according to *p*-value analysis and extremely changing as detected by our method. Two are directly associated with the bone remodeling process: Tenascin (P24821), an extracellular matrix protein, is upregulated in MSCs undergoing osteogenic differentiation [75] and contributes to extracellular matrix homeostasis through transforming growth factor beta (TGF- β ) pathway [76], while Fibrillin-1 (P35555) promotes osteoblast differentiation, also by interacting with TGF- β and bone morphogenetic proteins (BMPs) [77], and impairs osteoclastogenesis by sequestering TNF Superfamily Member 11 (TNFSF11/RANKL) [78]. On the other hand, PAICS (P22234) seems to be involved in de novo purine synthesis [79], and its specific role in bone homeostasis is still unclear. Interestingly, PAICS was the only protein also detected as extremely changing when comparing a doped bioactive glass, namely STe5, with the basal condition (i.e., no biomaterial also known as “plastic”), which might guide future research towards elucidating its effects related to biomaterial implantation.

Our method also identified some extremely changing proteins that are not detected through the classical analysis based on the *p*-value. While some showed no reported connection with biological processes explicitly associated with MSCs or the bone microenvironment, others might be of interest due to their known role in bone homeostasis. In particular, comparing STe5 with its undoped control, a TNC (P24821) and an isoform (Accession Number P24821-4) were detected as extremely changing, which are extracellular matrix components, together with Versican core protein (P13611) [80] that can bind TNC and regulate osteoblast differentiation [75], and inflammation or immunity [81]. Also, high mobility group protein B2 (P26583), involved in the osteoclastogenesis process and immune cell recruitment [82], was marked as displaying rare behavior. Similarly, comparing the content of EVs released by MSCs cultured in contact with AgSBA2 in comparison with the Control condition (Table 1), the expression of Type II collagen, present in cartilaginous matrix [83], was identified as extremely changing, while in the CuSBA3 vs Control (Table 2), alterations in Alpha-2-HS-glycoprotein (P02765), responsible for bone mineralization mostly in fetal tissues [84], was also evident in our analysis. Considering that the classical analysis could not highlight the aforementioned proteins as being relevant for further investigation, coupling both methods might lead to a more comprehensive overview of each biomaterial’s impact on MSC-EVs’ content.

In parallel, analyzing the differences in protein expression when comparing each one of the doped bioactive glasses with the basal condition “plastic” shows four proteins in common, all from the keratin family (KRT1, KRT2, KRT9, and KRT10). Generally, keratins are produced by epithelial cells, most often by keratinocytes present in the epidermis [85]. In fact, because MSCs can differentiate into keratinocytes in vitro [86], it has been exploited in concomitance with decellularized matrix from ovine small intestine submucosa tissue [87]. Our results show that the MSCs cultured in contact with any bioactive glass secrete EVs with a significantly inferior number of keratins than the basal condition, which can represent a lower tendency for differentiation into keratinocytes. Instead, given the increased expression of collagen IV α 3 chain in the silver- and copper-doped conditions, essential for cell-matrix binding in cartilage, but also skeletal muscle and bone in development [88,89], together with the upregulation of prelamin-A/C in tellurium-doped condition, are indicative of osteogenic differentiation [90].

Also, some identified proteins were not yet directly associated with the bioactivity of the musculoskeletal system; however, they might still be relevant in the context of biomaterial implantation. In the case of PTX (P26022), which is mostly known as a pro-inflammatory molecule released by several immune cell types, endothelial cells, and fibroblasts in response to interleukin (IL)-1 and tumor necrosis factor (TNF)- α , it appears to be downregulated in the tellurium-doped condition, which could indicate a differential immunomodulatory effect of MSCs depending on the presence of this bioactive glass. On the other hand, MSC culture in contact with STe5 leads to the release of EVs with higher content of CLSTN1, a regulator of axon branching and amyloid precursor protein trafficking and processing [91], in comparison with the basal condition, which might lead to neurogenesis stimulation (O94985).

Nevertheless, although the machine-learning method presented at this moment can provide complementary information to the classical analysis due to the pleiotropic nature of proteins, it is still essential to validate the postulated hypotheses through additional functional assays. Additional tables in Appendix C provide a functional context for the identified proteins.

### 3.2. Limitations of the Study

The methodology proposed in the current study presents some limitations because evaluating an anomaly involves selecting and adjusting a threshold for classifying instances as anomalies based on the characteristics of the specific problem. In this study, a statistical threshold has been selected, but this cut-off level might be adjusted according to the scientific goals and the nature of the research question.

Another consideration pertains to the usage of the Euclidean distance as a metric. Euclidean distance might be an appropriate choice in the case of a three-donor space, but it should be replaced when dealing with experiments involving more cell lines because it does not perform well on multidimensional spaces [92]. Also, the generalizability of the results should be managed carefully as it only refers to the actual donor space.

## 4. Materials and Methods

**Figure 4:**
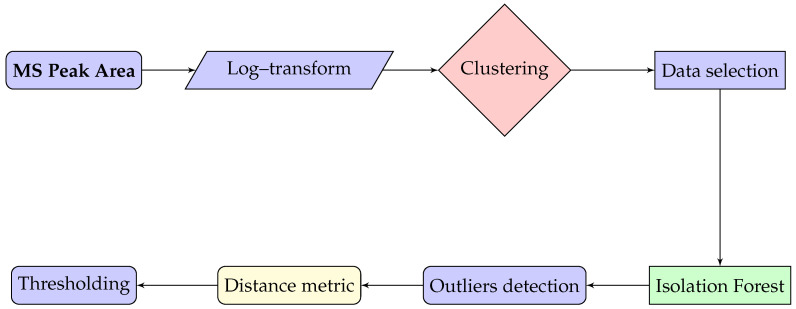
Scheme of the proposed methodology.

In the current investigation, the proposed analysis sequence summarized by Figure 4 is applied to evaluate three subject’s EV protein content of MSC cells cultured on top of different bioactive glasses detected through mass spectrometry. The MS dataset under investigation evaluated the normalized area reflecting the intensity of a mass spectral peak, which summarizes the number of ions contributing to that peak. It is proportional to the abundance of the ions with a specific mass-to-charge ratio in the sample, allowing for quantitative analysis. A larger peak area indicates a higher abundance of ions with that particular mass-to-charge ratio in the sample, while a smaller peak area indicates a lower abundance. The application of the suggested pipeline to a small sample real-world proteomic dataset with data coming from three donors will, on one side, demonstrate the usage of the methodology and, on the other, provide identification of unusual patterns indicative of protein activation on biomaterials.

### 4.1. Bioactive Glasses Preparation

In the present study, silica-based bioactive glasses, either doped on their surface with silver- or copper-ions via ion exchange or by including tellurium oxide directly to the glass as a substitute for silica, were prepared following the descriptions in [93,94,95]. The compositions of SBA2 and SBA3 (undoped controls) are shown in Table 9.

Both SBA2 and SBA3 were prepared using a melting and quenching process. In short, all the components listed above were melted in a platinum crucible at 1450 °C for 1 h, after which the melt was cooled in a brass mold to obtain glass bars with a diameter of 1 cm, which were then annealed at 500 °C for 13 h and cut into discs of 2 mm thickness. The discs were polished with SiC abrasive papers up to 1200 grit to level the surfaces. For the ion-doping procedure, silver (Ag^+^) and copper (Cu^2+^) ions were incorporated onto the surface of SBA2 and SBA3, respectively, through the ion-exchange process consisting of submerging them in an aqueous solution of AgNO_3_ (0.03 M) or Cu(CH_3_COO)_2_ (0.001 M) for 1 h, at 37 °C.

Additionally, the composition of ST with 5% molar tellurium or without (in previous works denominated as Ste0 or a doped version of STe5, [94]) is described in Table 10.

Likewise, ST bioactive glasses were also prepared by melt and quenching process, with a slightly modified protocol. In this case, the melting was done at 1500 °C for 1 h, while the annealing was performed at 550 °C for 13 h. All the samples were sterilized by heating at 100 °C for 3 h.

### 4.2. Mesenchymal Stem/Stromal Cell Isolation

MSCs were isolated from bone marrow samples of 3 donors affected by osteoarthritis who underwent joint replacement surgery. The samples were thoroughly mixed with 5–10 mL of Dulbecco’s Modified Essential Medium (DMEM) and passed through a 100 µm cell strainer into a 50 mL Falcon tube to remove any debris. Then, the cell mix was carefully overlaid in Lympholyte^®^ (Cedarlane, Burlington, ON, Canada) and centrifuged at 1100× *g*, for 30 min, with minimum acceleration and no brake. Then, the ring present in the interface was collected and washed with DMEM, centrifuging at 900× *g*, for 10 min. The cells were counted and plated at a concentration of 180,000 cells/cm^2^ in MSC growth medium (DMEM with low glucose supplemented with 10% heat-inactivated fetal bovine serum (FBS), 1% Penicillin/Streptomycin (P/S) and 0.5% Gentamycin). The cells were incubated at 37 °C, and the medium was changed twice per week until the cells reached approximately 80% confluence. For expansion, cells were treated with trypsin, counted, and transferred to a T75 tissue culture flask at a concentration of 2000 cells/cm^2^. Isolated cells were characterized according to the guidelines provided by the International Society for Cell and Gene Therapy (ISCT) [96]. MSCs between passages 3 and 6 were used for the experiments.

### 4.3. Wet-Lab Experimental Conditions

The experimental setup consisted of MSC culture obtained from three independent donors on several different bioactive glasses (and the respective Control conditions), using 5000 cells per disc at 37 °C, 5% CO_2_, for seven days. At the endpoint, the supernatants were collected for EV isolation through ultracentrifugation at 100,000× *g* for 2 h at 4 °C. The pellet enriched in EVs was then resuspended in 500 µL of Phosphate Buffer Saline (PBS 1×), and the EV protein content was evaluated through mass spectrometry. The initial data belonging to three donors contained the mass spectrum peak area from the samples of each participant. The following experimental conditions were tested:

- cell cultures on “SBA2”, “SBA3”, and “ST”, which are undoped bioactive glasses (i.e., controls or abbreviated as ctrl). Their composition was reported in Table 9 and Table 10.
- cell cultures on “SBA2”, “SBA3”, and “ST” modified bioactive glasses doped with silver, copper, and tellurium, respectively (i.e., doped).
- cell culture on “Plastic”, a baseline condition without the presence of biomaterials (i.e., plast)

The laboratory experiments aimed at establishing protein content modifications: those occurring between the doped glasses and the “plastic” condition could be a consequence of the presence of the bioactive glass. Furthermore, protein expression altered between the doped glasses and the respective control glass should be due to the metal ion doping [97]. Also, in vitro, the “Plastic” condition represents the absence of biomaterial, i.e., the physiological condition. Thus, comparing the doped conditions and the plastic parallels the comparison between normal tissue and implant. It should be remarked that there were no experimental differences between donors (culture conditions, number of wells, time-point, cell density, etc.).

### 4.4. Mass Spectrum Summary

Sample processing for MS analysis and data collection was conducted at the Mass Spectrometry unit of the University of Piemonte Orientale (Novara, Italy). EVs were lysed with RIPA buffer and sonicated. Proteins were precipitated with cold acetone, reduced in 25 μL of 100 mM NH_4_HCO_3_ with 2.5 μL of 200 mM DTT (Merck, Rahway, NJ, USA) at 60 °C for 45 min and then alkylated with 10 μL 200 mM iodoacetamide (Merck) for 1 h at RT in dark conditions. Digested peptides were analyzed with a UHPLC Vanquish system (Thermo Scientific, Rodano, Italy) coupled with an Orbitrap Q-Exactive Plus (Thermo Scientific, Rodano, Italy). Peptides were separated by a reverse phase column (Accucore™RP-MS 100 × 2.1 mm, particle size 2.6 μm). The column was maintained at a constant temperature of 40 °C at a flow rate of 0.200 mL/min. Mobile phase A and B were water and acetonitrile, respectively, both acidified with 0.1% formic acid. The analysis was performed using the following gradient: 0–5 min from 2% to 5% B; 5–55 min from 5% to 30% B; 55–61 from 30% to 90% B and hold for one minute, at 62.1 min the percentage of B was set to the initial condition of the run at 2% and hold for about 8 min in order to re-equilibrate the column, for a total run time of 70 min. The mass spectrometry analysis was performed in positive ion mode. The ESI source was used with a voltage of 2.8 kV. The capillary temperature, sheath gas flow, auxiliary gas, and spare gas flow were set at 325 °C, 45 arb, 10 arb, and 2, respectively. S-lens was set at 70 rf for the acquisition of spectra, and a data-dependent (ddMS2) top-10 scan mode was used. Survey full-scan MS spectra (mass range *m*/*z* 381 to 1581) were acquired with resolution R = 70,000 and AGC target 3 × 10^6^. MS/MS fragmentation was performed using high-energy c-trap dissociation (HCD) with resolution R = 35,000 and AGC target 1 × 10^6^. The normalized collision energy (NCE) was set to 30. The injection volume was 3 μL. The acquired raw MS data files were processed and analyzed using Proteome Discoverer with Chimerys (v3.0.0.757, Thermo Fisher Scientific). SequestHT was used as a search engine, and the following parameters were chosen. Database: Homo sapiens (Uniprot, downloaded on 1 February 2018) enzyme: trypsin; max. missed cleavage sites: 2; static modifications: carbamidomethyl (C); dynamic modifications: oxidation (M); precursor mass tolerance: 10 ppm; fragment mass tolerance: 0.02 Da. Only peptides and proteins with FDR value < 0.01 were reported (fixed cut-off). An abundance of identified peptides was determined by label-free quantification (LFQ) using match between runs.

### 4.5. Computational Resources

All numerical experiments of the proposed analysis pipeline were demonstrated on commodity hardware: a Dynabook (Tokyo, Japan) laptop computer equipped with an Intel i5 CPU and 16 GB RAM. This choice ensured the reproducibility of the current analysis sequence by other groups or researchers because it does not require intensive resources such as cloud or cluster computing.

### 4.6. Dry-Lab Experimental Sequence

The proposed workflow, starting from the raw mass spectrum peak area, involved the following steps:

1. The raw values from the three donors were log-transformed
2. The log-transformed values were clustered, and the values of the same cluster were taken to ensure analysis of similar data representing the same biological phenomena
3. Each value was labeled as outlier (potential “anomaly” or extreme variation) or not applying Isolation Forest
4. Computed the distance between outlier proteins in the donors’ 3D space to identify abnormal variations in the EV-related protein expression

#### 4.6.1. Proposed Sequence: Log-Transformation Preprocessing

The log transformation of the raw peaks was performed because mass spectrometry data can have a wide range of intensities, and some peaks might be much larger than others due to various factors such as instrument variability, sample concentration, and ionization efficiency. Indeed, log transformation helps normalize the data by compressing the dynamic range and making smaller peaks more visible. Additionally, log transformation can reduce the impact of random noise in the data. Noise often contributes more to the lower intensity peaks, and by taking the logarithm, the noise is dampened, making it easier to distinguish valid signals from noise.

#### 4.6.2. Proposed Sequence: Clustering

OPTICS (Ordering Points To Identify the Clustering Structure) is a data clustering algorithm used in machine learning to identify natural clusters and their hierarchies in a dataset [98]. It is handy for datasets with varying densities, irregular shapes, and noise. OPTICS is an extension of the DBSCAN (Density-Based Spatial Clustering of Applications with Noise) algorithm [99], which aims to discover clusters based on the density of data points.

In OPTICS, two main parameters are to be evaluated: core (minPts) and reachability distances (also called ϵ parameter). The concept of “reachability distance” means that for a data point P, the reachability distance to another data point Q is defined as the maximum distance between P and Q, such that P can be directly reached from Q while staying within a predefined neighborhood size. Instead, a data point’s core distance is the smallest such that there are at least a certain number of points within that distance, forming a dense region around the point. In the current investigation, a reachability parameter of 0.05 and a minPts parameter of 50 were applied.

**Figure 5:**
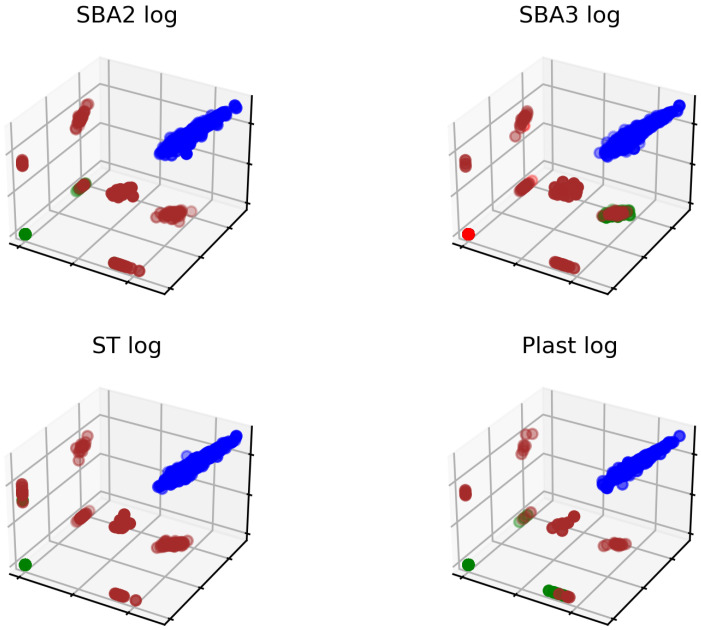
All data from the three donors and each experimental condition underwent automatic labeling to store only uniformly distributed values: the points in the blue cluster were retained in the next steps of the experimental sequence. The other colors represent the additional clusters identified by OPTICS.

**Figure A5:**
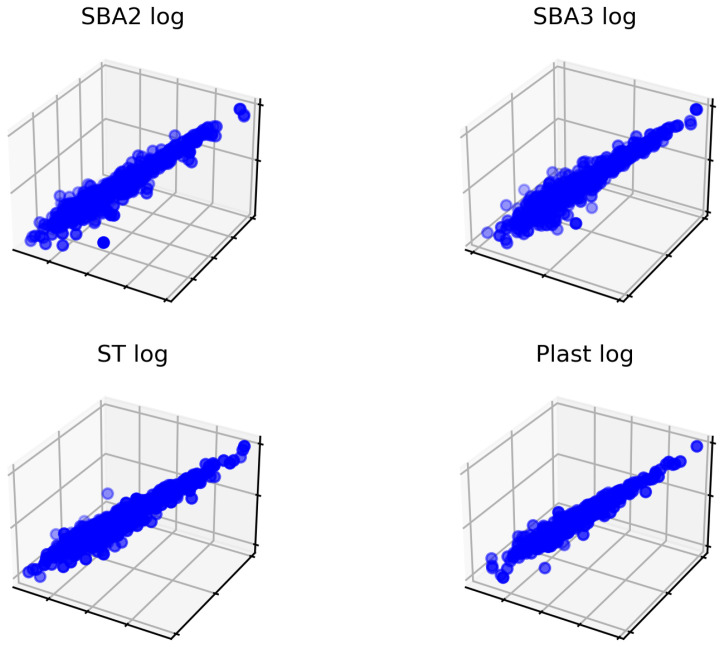
Protein expression values from the three subjects belonging to the blue cluster retained for further analysis.

The effect of clustering all log-transformed peak values is shown in Figure 5. By employing only values found in the blue cluster, the analysis focused on finding aberrant proteins inside a group with similar expression profiles, excluding proteins markedly belonging to other clusters that probably portray different biological phenomena. Indeed, among the different biological activities depicted in Figure 5, the values inside the blue cluster might represent the most relevant biological organization, also because peak areas were positive in all three donors (Figure A5). When negative peaks appear in the mass spec chromatogram, particularly in the total or extracted ion chromatogram, it could be attributed to various factors. One possible explanation could be compounds with higher proton affinity co-eluting.

In proteomics, clustering methods such as hierarchical clustering or k-means clustering are often applied to identify groups of proteins with similar expression patterns or functional relationships. However, on our dataset, OPTICS performed better than classic clustering methods; this tendency is also confirmed in other sources from the literature [100].

#### 4.6.3. Proposed Sequence: Outlier Detection by Isolation Forest

Isolation Forest is a machine-learning algorithm for anomaly detection and outlier identification [101]. Isolation Forest conceptualizes that anomalies are usually rare instances that can be “isolated” more quickly than regular instances. The algorithm constructs a binary tree-like structure in which each internal node represents a feature and a split point. In contrast, each leaf node represents an isolated instance or an anomaly. To detect anomalies, the algorithm calculates the path length from the root of the tree to the leaf where a data point resides. Anomalies are expected to have shorter paths because they are isolated more quickly. The average path length of a data point across all trees in the forest is used as a score of atypical expression. Smaller average path lengths indicate higher anomaly scores.

**Figure 6:**
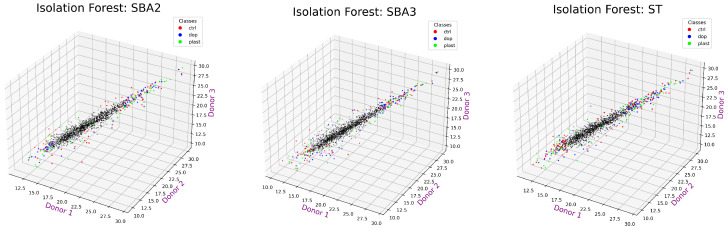
Values from the three donors marked as black dots were considered inliers, thus close to each other, in the three experimental conditions (Plast, Control, and Doped) by the Isolation Forest algorithm, whereas colored points were those showing more relevant changes (aka outliers).

As depicted in Figure 6, the Isolation Forest identified a set of core values in the distribution that are close to each other: these values were marked as black dots and could be considered those with similar peak areas. To find proteins showing extremely changing behavior between experimental conditions, only the colored values were retained for being considered outliers by the algorithm. Among the outliers, the possibility of finding proteins with unusual expression in the three experimental conditions might be high. Please note that the “plast” condition was the same in all three graphs.

**Figure 7:**
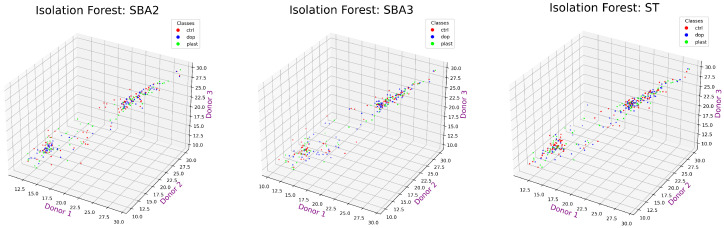
Retaining only the values considered outliers by Isolation Forest. The three axes represent the three donors.

Figure 7 reports only the values of protein expression kept for further analysis.

#### 4.6.4. Proposed Sequence: Distance Metric

**Figure A6:**
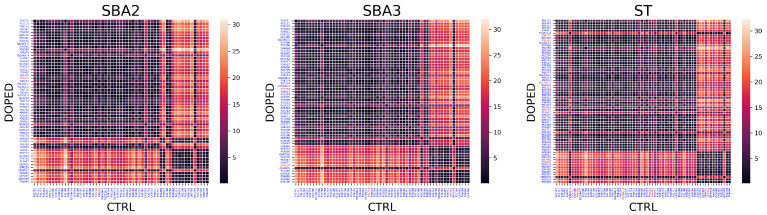
Heatmap with Euclidean distances between the Control and Doped conditions. The left panel is “SBA2” condition, the central image the “SBA3”, whereas the right one is “ST”.

**Figure A7:**
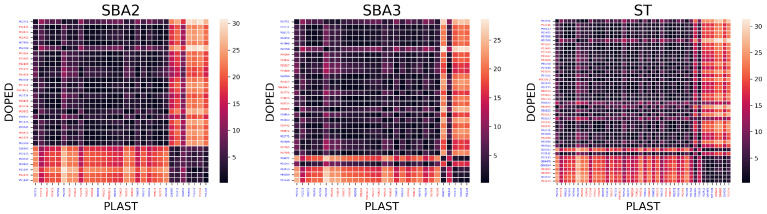
Heatmap with Euclidean distances between the Plastic and Doped conditions. The left panel is “SBA2” condition, the central image the “SBA3”, whereas the right one is “ST”.

Euclidean distance measures the straight-line distance between two or three points in Euclidean space; in mathematics, Euclidean space refers to the geometric space of classical Euclidean geometry, the familiar two- and three-dimensional space where the Pythagorean theorem holds. Euclidean distance is often used to quantify the similarity or dissimilarity between two points in space. In machine learning, Euclidean distance is commonly used in clustering, classification, and nearest-neighbor algorithms to measure the distance between data points. Figure A6 summarizes all distances between proteins in the Control (*x*-axis) versus Doped (*y*-axis) conditions. Figure A7 shows distances between protein expression in the Plastic (*x*-axis) versus Doped (*y*-axis) conditions. Lighter values display higher distance, meaning dissimilarity. The variations measured by the Euclidean distance for the same type of protein are the diagonal of the matrix shown in Figure A6 and Figure A7. These distances were all gathered and thresholded to classify instances as anomalies. The threshold was the mean plus a standard deviation. The mean plus one standard deviation is a measure that provides information about the spread or dispersion of a set of data in a normal distribution. In a normal distribution, approximately 68% of the data falls within one standard deviation of the mean [102].

**Figure 8:**
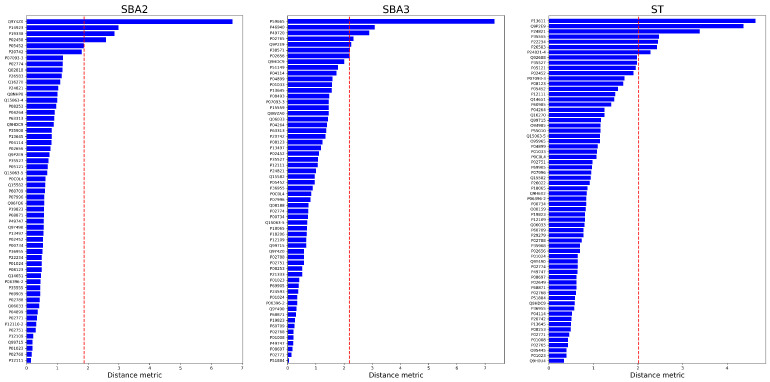
Euclidean distances between the Control and Doped conditions. The left panel is “SBA2” condition, the central image the “SBA3”, whereas the right one is “ST”. The statistical threshold is the red vertical line.

**Figure 9:**
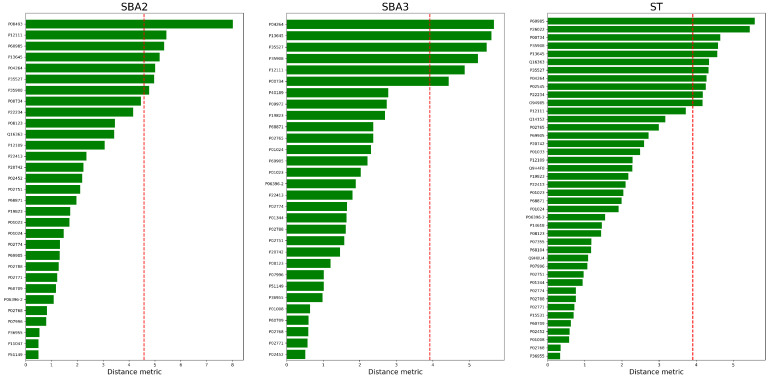
Euclidean distances between the Plastic and Doped conditions. The left panel is “SBA2” condition, the central image the “SBA3”, whereas the right one is “ST”. The statistical threshold is the red vertical line.

The Figure 8 and Figure 9 collect the sorted Euclidean distances as bar plots. The threshold is represented by the vertical dashed line. Only the proteins above the threshold were identified as “anomalies” and kept for final evaluation. During the procedure, a single threshold has been selected as a boundary rule to classify a protein as aberrant or not based on Euclidean distance. However, each distance could be evaluated individually because some appear more prominent than others, and natural gaps are observable in the bars of Figure 8 and Figure 9.

It should be remarked that the anomaly detection sequence proposed in the current study targets three-donor experiments and employs the L2-norm as a metric to judge the outliers. Experiments involving large cohorts of subjects might replace the Euclidean metric with a different distance function better suited to address the “curse of dimensionality”, as it might lead to inconsistencies on high-dimensional data [103]. However, it has been found that for noncentral t-distributions, the Euclidean distance remains highly effective even when dealing with higher dimensions.

## 5. Conclusions

In the case of a limited sample size, statistics should be managed accurately. The current study proposed an analysis sequence to evaluate protein expression from the actual values of the subjects involved in an experiment, exploiting machine-learning anomaly detection techniques. The proposed procedure might support and assist researchers in assessing findings when employing small experimental datasets by finding abnormal protein behavior in the data. Attracting the researcher’s attention to individual proteins in different ways compared to standard statistical testing can provide additional evidence or reveal hidden aspects of the experimental design. The methodology has been verified in an experimental setting where the EV protein content of MSC cultured on three bioactive glasses, doped or not with metallic ions, has been investigated. The procedure identified a subset of proteins that showed highly changing behavior between experimental conditions: the effect of ion doping is described by a peculiar set of abnormal proteins each metal activates. Conversely, comparing doped biomaterials and the baseline plastic scaffold involved a mutual set of proteins. Some proteins were significant at the *t*-test, and others had a high variance pattern between experimental conditions. The additional knowledge of the data under exam offered by this technique might provide further understanding of the experimental setting and the outcomes it provides.

## Figures and Tables

**Table 1:** Most expressed proteins in Control or Silver doped bioactive glass.

Accession Number	Gene Name	Protein Name
P02458	*COL2A1*	Collagen alpha-1(II) chain
P19338	*NCL*	Nucleolin
P14923	*JUP*	Junction plakoglobin
Q9Y4Z0	*LSM4*	U6 snRNA-associated Sm-like protein LSm4

**Table 2:** Most expressed proteins in Control or Copper-doped bioactive glass.

Accession Number	Gene Name	Protein Name
P02656	*APOC3*	Apolipoprotein C-III
P38571	*LIPA*	Lysosomal acid lipase/cholesteryl ester hydrolase
Q9P2E9 **	*RRBP1*	Ribosome-binding protein 1
P02765	*AHSG*	Alpha-2-HS-glycoprotein
P49720	*PSMB3*	Proteasome subunit beta type-3
P46940	*IQGAP1*	Ras GTPase-activating-like protein IQGAP1
P59665	*DEFA1*	Neutrophil defensin 1

** More than 95% probability the observed data are inconsistent with the null hypothesis (*t*-test *p* < 0.05).

**Table 3:** Most expressed proteins in Control or Tellurium-doped bioactive glass.

Accession Number	Gene Name	Protein Name
P24821-4	*TNC*	Isoform 4 of Tenascin
P26583	*HMGB2*	High mobility group protein B2
P22234 *	*PAICS*	Multifunctional protein ADE2
P35555 **	*FBN1*	Fibrillin-1
P24821 *	*TNC*	Tenascin
Q9P2E9 **	*RRBP1*	Ribosome-binding protein 1
P13611	*VCAN*	Versican core protein

** More than 95% probability the observed data are inconsistent with the null hypothesis (*t*-test *p* < 0.05). * Between 90% and 95% probability, the observed data are inconsistent with the null hypothesis (*p*-values of the *t*-test between 0.05 and 0.1).

**Table 4:** Most expressed proteins in Plastic or Silver doped bioactive glass.

Accession Number	Gene Name	Protein Name
P35908 **	*KRT2*	Keratin, Type II cytoskeletal 2 epidermal
P35527 **	*KRT9*	Keratin, Type I cytoskeletal 9
P04264 **	*KRT1*	Keratin, Type II cytoskeletal 1
P13645 **	*KRT10*	Keratin, Type I cytoskeletal 10
P60985 *	*KRTDAP*	Keratinocyte differentiation-associated protein
P12111 **	*COL6A3*	Collagen alpha-3(VI) chain
P08493	*MGP*	Matrix Gla protein

** More than 95% probability the observed data are inconsistent with the null hypothesis (*t*-test *p* < 0.05). * Between 90% and 95% probability, the observed data are inconsistent with the null hypothesis (*p*-values of the *t*-test between 0.05 and 0.1).

**Table 5:** Most expressed proteins in Plastic or Copper-doped bioactive glass.

Accession Number	Gene Name	Protein Name
P00734 **	*F2*	Prothrombin
P12111 *	*COL6A3*	Collagen alpha-3(VI) chain
P35908 **	*KRT2*	Keratin, Type II cytoskeletal 2 epidermal
P35527 **	*KRT9*	Keratin, Type I cytoskeletal 9
P13645 **	*KRT10*	Keratin, Type I cytoskeletal 10
P04264 **	*KRT1*	Keratin, Type II cytoskeletal 1

** More than 95% probability the observed data are inconsistent with the null hypothesis (*t*-test *p* < 0.05). * Between 90% and 95% probability, the observed data are inconsistent with the null hypothesis (*p*-values of the *t*-test between 0.05 and 0.1).

**Table 6:** Most expressed proteins in Plastic or Tellurium-doped bioactive glass.

Accession Number	Gene Name	Protein Name
O94985 **	*CLSTN1*	Calsyntenin-1
P22234 **	*PAICS*	Multifunctional protein ADE2
P02545 *	*LMNA*	Prelamin-A/C
P04264 **	*KRT1*	Keratin, Type II cytoskeletal 1
P35527 **	*KRT9*	Keratin, Type I cytoskeletal 9
Q16363	*LAMA4*	Laminin subunit alpha-4
P13645 **	*KRT10*	Keratin, Type I cytoskeletal 10
P35908 **	*KRT2*	Keratin, Type II cytoskeletal 2 epidermal
P00734 **	*F2*	Prothrombin
P26022 *	*PTX3*	Pentraxin-related protein PTX3
P60985 **	*KRTDAP*	Keratinocyte differentiation-associated protein

** More than 95% probability the observed data are inconsistent with the null hypothesis (*t*-test *p* < 0.05). * Between 90% and 95% probability, the observed data are inconsistent with the null hypothesis (*p*-values of the *t*-test between 0.05 and 0.1).

**Table 7:** Within-group variance in the ctrl and doped experimental conditions.

Experiment	Protein	*p*-Value	Ctrl Var.	Doped Var.	Abs Var. *
SBA2	P02458	0.77209	1.27	2.26	0.99
SBA2	P19338	0.7934	2.76	0.16	2.6
SBA2	P14923	0.90066	3.85	1.55	2.3
SBA2	Q9Y4Z0	0.37355	13.0	0.72	12.28
SBA3	P02656	0.19829	0.73	0.18	0.55
SBA3	P38571	0.85016	0.21	2.61	2.4
SBA3	Q9P2E9	0.03166	0.22	0.08	0.14
SBA3	P02765	0.51954	1.8	0.2	1.6
SBA3	P49720	0.80732	1.44	1.55	0.11
SBA3	P46940	0.67908	0.51	1.66	1.15
SBA3	P59665	0.40182	8.47	2.79	5.68
ST	P24821-4	0.4643	1.02	1.73	0.71
ST	P26583	0.20189	0.1	0.71	0.61
ST	P22234	0.08797	0.5	0.17	0.33
ST	P35555	0.04938	0.22	0.34	0.12
ST	P24821	0.05846	0.13	0.96	0.83
ST	Q9P2E9	0.03078	0.32	0.23	0.09
ST	P13611	0.16264	1.83	5.37	3.54

* Absolute difference between variances.

**Table 8:** Within-group variance in the plast and doped experimental conditions.

Experiment	Protein	*p*-Value	Ctrl Var.	Doped Var.	Abs Var. *
SBA2	P35908	0.00245	0.07	0.28	0.21
SBA2	P35527	0.00029	0.02	0.14	0.12
SBA2	P04264	0.00015	0.01	0.25	0.24
SBA2	P13645	0.00041	0.02	0.35	0.33
SBA2	P60985	0.08047	0.27	0.84	0.57
SBA2	P12111	0.00145	0.54	0.04	0.5
SBA2	P08493	0.10463	2.05	1.65	0.4
SBA3	P00734	0.0291	0.25	0.01	0.24
SBA3	P12111	0.05029	0.54	0.63	0.09
SBA3	P35908	0.00252	0.07	0.57	0.5
SBA3	P35527	0.00038	0.02	0.53	0.51
SBA3	P13645	0.00059	0.02	0.79	0.77
SBA3	P04264	0.00018	0.01	0.66	0.65
ST	O94985	0.02078	0.01	0.26	0.25
ST	P22234	0.03636	1.17	0.17	1.0
ST	P02545	0.08999	2.02	0.17	1.85
ST	P04264	0.00015	0.01	0.07	0.06
ST	P35527	0.00079	0.02	0.29	0.27
ST	Q16363	0.25842	2.89	1.69	1.2
ST	P13645	0.00037	0.02	0.07	0.05
ST	P35908	0.00244	0.07	0.16	0.09
ST	P00734	0.02785	0.25	0.03	0.22
ST	P26022	0.08427	0.41	2.51	2.1
ST	P60985	0.04855	0.27	0.69	0.42

* Absolute difference in variance.

**Table 9:** Nominal compositions of SBA2 and SBA3 control bioactive glasses.

mol.%	SiO_2_	Na_2_O	CaO	P_2_O_5_	B_2_O_3_	Al_2_O_3_
SBA2	48	18	30	3	0.43	0.57
SBA3	48	26	22	3	0.43	0.57

**Table 10:** Nominal compositions of the ST control bioactive glass and ST after ion doping.

mol.%	SiO_2_	Na_2_O	CaO	P_2_O_5_	TeO_2_
STe0	48.6	16.7	34.2	0.5	0
STe5	43.6	16.7	34.2	0.5	5

**Figure A1:**
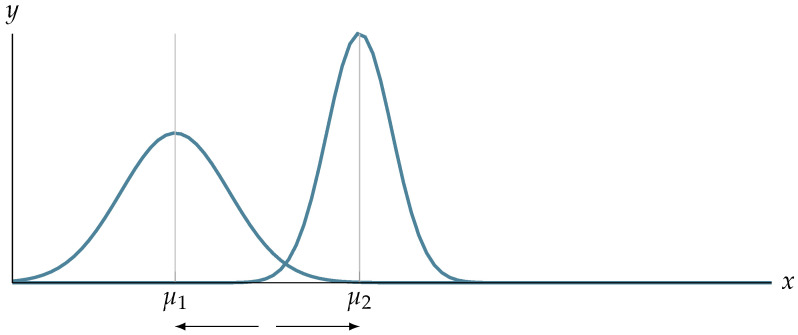
Distributions of the values from the three donors in Table A1 (Protein GAMMA). There is a statistically significant difference between experimental conditions, exemplified by the distance between averages.

**Figure A2:**
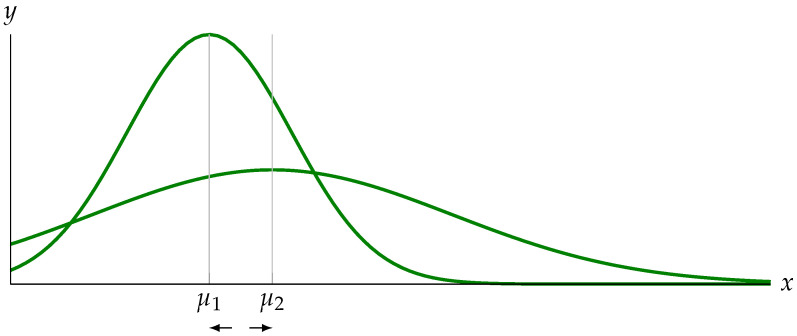
Distributions of the values from the three donors in Table A2 (Protein OMEGA). There is no significant difference between means in the two experimental conditions.

**Figure A3:**
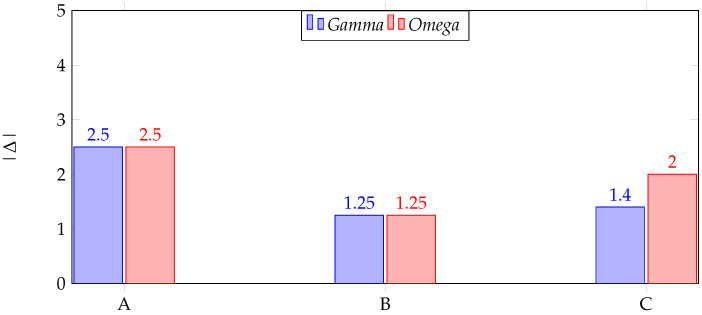
Hypothetical (absolute) differential expression of the proteins GAMMA and OMEGA in the three donors “A”, “B”, and “C”.

**Figure A4:**
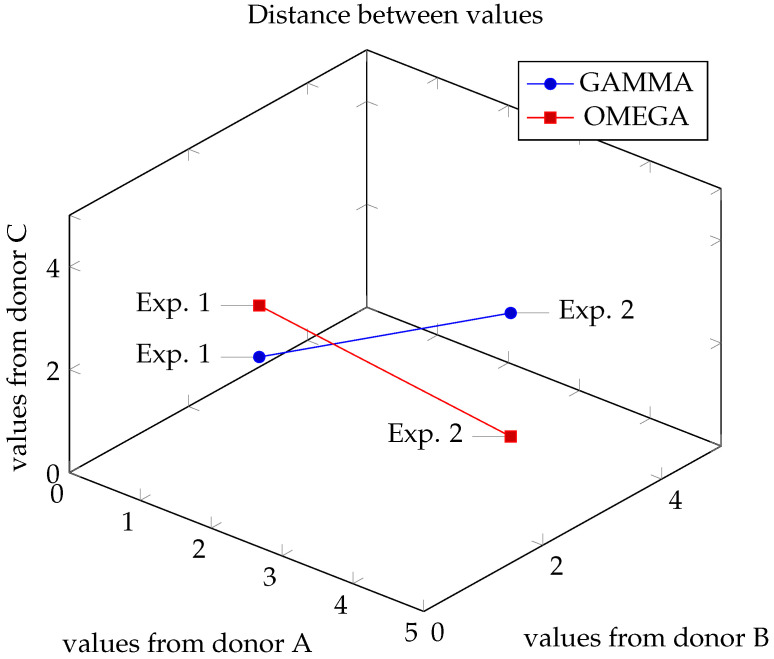
Example of the distance calculation between two points, each one representing an experimental condition in the 3D subjects’ space.

## Data Availability

The data presented in this study are available on request from the corresponding author.

## Abbreviations

CPU	Central Processing Unit
Ctrl	Control bioactive glasses
CV	Coefficient of Variation
BMP	Bone Morphogenetic Proteins
DMEM	Dulbecco’s Modified Essential Medium
EV	Extracellular Vesicles
ISCT	International Society for Cell and Gene Therapy
ML	Machine Learning
MSC	Mesenchymal Stem Cells
MS	Mass Spectrometry
Plast	Experimental condition on Plastic material (baseline)
RAM	Random Access Memory
SBA2	Experimental setting involving bioactive glass doped or not with silver
SBA3	Experimental setting involving bioactive glass doped or not with copper
ST	Experimental setting involving bioactive glass doped or not with tellurium

## Appendix A. Theoretical Proteomic Scenario

This section analyzes theoretical proteomic small sample data, describing the effect of statistics on the hypothetical proteins called Alpha and Omega. Furthermore, applying a similarity measure, such as the Euclidean distance, will be shown. For example, one might use statistical tests in small sample datasets to make inferences about population parameters. At the same time, distance functions can provide insights into the local relationships and patterns within the data.

### Appendix A.1. A Toy Example on Proteomics’ Small Sample Dataset

This paragraph reports a theoretical case scenario of diverging patterns in protein expression between experimental conditions to give a practical example of the situations one could face in the case of a small proteomic dataset from three donors. For instance, an experimental setup collected data representing the protein expression levels of three donors (“A”, “B”, and “C”) in two laboratory conditions (Exp. 1 and Exp. 2). A GAMMA hypothetical protein expression in the two experiments is reported in Table A1.

**Table A1:** Theoretical protein expression in three donors (Protein GAMMA).

Donor	Exp. 1	Exp. 2	Δ ^1^	
A	1	3.5	2.5	
B	2	3.25	1.25	
C	1.5	2.9	1.4	
	1.5	3.2	1.71	Mean (μ)
	0.5	0.3	0.68	St. Dev. (σ)

^1^ Difference between experimental conditions.

According to the Shapiro–Wilk Test (*p* = 0.3621), both distributions conform to normalcy, and no outliers are detected with the Tukey Fence test (*k* = 1.5); thus, the next step in a hypothetical analysis could be verifying statistically if the experimental conditions produced diverging outcomes. A usual choice could be the two-tailed *t*-test attempting to verify wherever:

- $H_{0}:\mu_{1}=\mu_{2}$
- $H_{A}:\mu_{1}\neq \mu_{2}$

using the formula $t=\frac{\mu_{\Delta }}{\frac{\sigma_{\Delta }}{\sqrt{n}}}$ . The $H_{0}$ represents the null hypothesis (in simple words, “nothing is going on” [104]), whereas the alternative hypothesis is often the one an investigator hopes to advance. From the data collected in Table A1, the *t*-test will appear statistically significant ( p=0.0489 , t=4.3564 , df=2 ), therefore implying the rejection of the $H_{0}$ and the acceptance of $H_{A}$ . Figure A1 shows the two hypothetical distributions and highlights the difference between averages that could be interpreted as not given by chance.

During the same theoretical laboratory experiment, the levels recorded in another protein called OMEGA were collected in Table A2. In this case, donor “C” levels are markedly under-expressed in the second experimental condition, resulting in a *t*-test not significant ( p=0.7060 , t=0.4350 , df=2 ). Also, in this theoretical situation, distributions are assumed to be normal (Shapiro–Wilk Test with *p* = 0.9101, no outliers found with the Tukey Fence method).

**Table A2:** Theoretical protein expression in three donors (Protein OMEGA).

Donor	Exp. 1	Exp. 2	Δ ^1^	
A	1	3.5	2.5	
B	2	3.25	1.25	
C	2.5	0.5	−2	
	1.83	2.41	0.58	Mean (*μ*)
	0.76	1.66	2.32	St. Dev. (*σ*)

^1^ Difference between experimental conditions.

The theoretical distributions of the OMEGA protein levels in the two experiments are illustrated in Figure A2. The two shapes overlap consistently with averages close to each other.

Observing the absolute value difference in the two experimental conditions as shown in Figure A3, one could argue the under-expression for protein OMEGA was not detected statistically due to the negative shift caused by donor “C” on the average values of the second experiment.

Statistical power is the probability that a statistical test will reject a false null hypothesis correctly. In other words, it is the likelihood of detecting a true effect if it exists. In the context of small samples, statistical power can be particularly challenging because smaller sample sizes may have less ability to detect actual effects. With such small samples as found in this toy example, statistical power is typically low, making it difficult to detect true effects even if they exist. Indeed, the theoretical results of protein GAMMA might be exaggerated (Cohen’s *d* = 2.5152) propounding for a cautionary interpretation of the statistical results, whereas for protein OMEGA Cohen’s *d* was 0.2511.

### Appendix A.2. Proposed Alternative Data Evaluation Method

Another way to study the magnitude of the changes could be by calculating the distance between observations as shown in Figure A4. The axes represent each subject “A”, “B”, and “C” expression levels for the proteins GAMMA and OMEGA of Table A1 and Table A2. The lines joining the coordinates are the Euclidean distances between observations. The advantage of considering distances between actual values is to move from a three-dimensional subject’s space to a one-dimensional vector of distances describing the shift between values in the two experimental conditions for each protein.

A distance metric, also known as a distance function or similarity measure, is a mathematical function that quantifies the “distance” or “closeness” between two objects in a space. These descriptors define the similarity or dissimilarity between data points, providing a basis for comparing and analyzing their relationships. The Euclidean distance protein GAMMA distance was 3.126, whereas OMEGA was 3.437. Despite the *t*-test not finding a significant difference between experimental conditions in OMEGA, the distance function could report that the changes in three donors merit further attention.

## Appendix B. Additional Definitions and Images

This section collects general definitions, explanations, and additional figures or tables.

### Appendix B.1. Mass Spectrometry

Mass spectrometry allows researchers to determine the mass-to-charge ratio of ions, which provides information about the composition and structure of molecules, including proteins [105]. To analyze proteins using mass spectrometry, they are typically digested into smaller peptide fragments, often done using enzymes such as trypsin. Peptides are more amenable to mass spectrometry analysis because of their smaller size. The digested peptide fragments are introduced into the mass spectrometer after being converted into gas-phase ions. The ionization process is typically done through various ionization techniques, such as electrospray or matrix-assisted laser desorption/ionization. Once ionized, the peptides are subjected to mass analysis to measure the ions’ mass-to-charge ratio (*m*/*z*). The resulting data are presented as a mass spectrum, showing ions’ intensity at different *m*/*z* values. The obtained mass spectra are compared to databases of known protein sequences to determine the proteins’ identity in the sample based on the masses of the peptides and their sequence information (aka peptide identification) [106]. In addition to identification, mass spectrometry can also be used to quantify proteins by comparing the intensity of peptide ions between different samples, which can provide insights into the relative abundance of specific proteins.

### Appendix B.2. Outliers

An outlier is an observation or data point that significantly differs from the majority of other observations in a dataset: outliers are data points that deviate substantially from the overall pattern or distribution of the rest of the data; they are often located far from the mean or median of the dataset. Outliers can be found in various data types and impact the results of statistical analyses. Outliers can strongly influence statistical measures such as the mean and standard deviation; therefore, outliers can significantly skew these measures [107]. They also could represent unusual or rare events, errors in data collection, or extreme values that are genuinely part of the data distribution. Indeed, outliers can result from genuine variability in the data, but they also could be measurement errors or anomalies in the data collection process [108]. For this reason, understanding the causes of outliers is important for deciding how to handle them [109]. Common types of outliers could be:

- outliers impacting the entire dataset and characterized by extreme values that deviate from the overall pattern of the data. They can significantly affect summary statistics and models being usually referenced as global outliers
- points considered unusual or extreme within a specific subgroup or context may not be outliers when the entire dataset is considered. Detecting these types of points requires considering subsets of the data, and are called contextual outliers.
- Positional outliers are outliers that deviate from the typical position within a distribution. These are identified based on their location in relation to measures such as the mean, median, or quartiles.
- Outliers that cause the distribution of the data to be skewed; they can have a substantial impact on the shape of the distribution, making it asymmetrical (aka skewed outliers).
- Groups of observations that collectively deviate from the expected pattern may not be identified when examining individual data points but become apparent when considering groups of observations (aka collective outliers).
- In multivariate analysis, observations that deviate from the overall pattern in a multidimensional space may be considered outliers.
- Masked outliers are not immediately apparent in univariate analysis but become noticeable when considering interactions or relationships between variables. These outliers may affect the results of statistical models.
- Unexpected events or changes in the underlying process generating the data might produce novel occurrences that could be marked as outliers (i.e., innovational outliers).
- Points with values higher or lower than the majority of the data significantly impact the mean and standard deviation (i.e., additive outliers).

Outlier detection is a complex process, as numerous types of outliers may exist in a dataset; the attributes of the data may have intricate dependencies on each other, and there is no limit to the possible ways in which they could interact.

### Appendix B.3. Protein Expression Points Selected by Clustering

Clustering in proteomic expression levels can be a powerful technique for selecting data subsets based on similarities or patterns in protein expression. The OPTICS algorithm identified strictly positive values belonging to one cluster, ensuring an unsupervised data selection focusing on samples within the blue cluster that could exhibit similar protein expression patterns or represent closer biological conditions or phenotypes.

### Appendix B.4. Matrices of Euclidean Distances

When it comes to generating heatmaps for proteomics, the Euclidean distance is a widely used method, which is often considered to be a standard. This distance metric is based on the straight-line distance between two points, and it is used to calculate the similarity or dissimilarity between different samples or variables. Generally, this measure is favored because it is easy to compute and interpret, and it provides a good balance between sensitivity and robustness [110].

### Appendix B.5. Mean Variability in Each Experiment

**Table A3:** Mean variance on all experiments from Table 7 and Table 8.

Biomaterial	Mean Var. Ctrl	Mean Var. Doped	Mean Abs Diff.
SBA2	5.22	1.17	4.54
SBA3	1.91	1.3	1.66
ST	0.59	1.36	0.89
**Biomaterial**	**Mean Var. Plast**	**Mean Var. Doped**	**Mean Abs Diff.**
SBA2	0.43	0.51	0.34
SBA3	0.15	0.53	0.46
ST	0.65	0.56	0.68

**Table A4:** Mean CV on all experiments.

Biomaterial	Mean CV Ctrl	Mean CV Doped	Mean Abs Diff.
SBA2	17.563	8.594	8.969
SBA3	8.442	8.394	0.048
ST	5.388	7.591	2.203
**Biomaterial**	**Mean CV Plast**	**Mean CV Doped**	**Mean Abs Diff.**
SBA2	3.1	4.271	1.171
SBA3	1.555	3.485	1.929
ST	4.359	4.262	0.097

## Appendix C. Gene Ontology Enrichment Analysis

Gene ontology enrichment analysis was included to assess the functional significance of the sets of abnormal proteins in each experimental condition. The following tables report the biological processes, molecular functions, and cellular components associated with the given protein list in homo sapiens.

**Table A5:** Ctrl or silver doped bioactive glass.

Ontology	Accession	GO Class
Biological Process	-	-
Molecular Function	GO:0042731	PH domain binding
Cellular Component	GO:0001533	Cornified envelope
	GO:0099080	Supramolecular complex

**Table A6:** Ctrl or copper-doped bioactive glass.

Ontology	Accession	GO Class
Biological Process	GO:0071830	triglyceride-rich lipoprotein particle clearance
Molecular Function	-	-
Cellular Component	-	-

**Table A7:** Ctrl or tellurium-doped bioactive glass.

Ontology	Accession	GO Class
Biological Process	-	-
Molecular Function	-	-
Cellular Component	GO:0099535	synapse-associated extracellular matrix
	GO:0005788	endoplasmic reticulum lumen

**Table A8:** Plast or silver doped bioactive glass.

Ontology	Accession	GO Class
Biological Process	GO:0051291	protein hetero-oligomerization
	GO:0018149	peptide cross-linking
	GO:0045684	positive regulation of epidermis development
	GO:0045103	intermediate filament-based process
	GO:0097435	supramolecular fiber organization
	GO:0009888	tissue development
	GO:0030855	epithelial cell differentiation
	GO:0048513	organogenesis
Molecular Function	GO:0005198	structural molecule activity
Cellular Component	GO:0001533	cornified envelope
	GO:0099080	supramolecular complex
	GO:0045111	intermediate filament cytoskeleton
	GO:0062023	collagen-containing extracellular matrix
	GO:1903561	extracellular vesicle
	GO:0005615	extracellular space

**Table A9:** Plast or copper-doped bioactive glass.

Ontology	Accession	GO class
Biological Process	GO:0051291	protein hetero-oligomerization
	GO:0018149	peptide cross-linking
	GO:0042730	fibrinolysis
	GO:0045684	positive regulation of epidermis development
	GO:0045103	cytoskeleton intermediate filament process
	GO:0097435	supramolecular fiber organization
	GO:0008544	epidermis development
	GO:0030855	epithelial cell differentiation
	GO:0043588	skin development
Molecular Function	GO:0005198	structural molecule activity
Cellular Component	GO:0001533	cornified envelope
	GO:0099080	macromolecular complex
	GO:0045111	intermediate filament cytoskeleton
	GO:0005788	endoplasmic reticulum lumen
	GO:0062023	collagen-containing extracellular matrix
	GO:0043230	extracellular organelle
	GO:0031982	extracellular vesicle
	GO:0005615	extracellular space

**Table A10:** Plast or tellurium-doped bioactive glass.

Ontology	Accession	GO class
Biological Process	GO:0018149	peptide cross-linking
	GO:0045103	cytoskeleton intermediate filament-based process
	GO:0008544	epidermis development
Molecular Function	GO:0005198	structural molecule activity
Cellular Component	GO:0001533	cornified envelope
	GO:0099081	supramolecular polymer
	GO:0045111	intermediate filament cytoskeleton
	GO:0030312	external encapsulating structure
	GO:0043230	extracellular organelle
	GO:0031982	extracellular vesicle
	GO:0005615	extracellular space

The statistical analysis tool selected was PANTHER overrepresentation test (version 18.0), whereas the annotation software was https://zenodo.org/records/10536401, 15 November 2023. Fisher’s exact test was performed with FDR correction.
