# Correction: Nascimben et al. Extracellular Vesicle Protein Expression in Doped Bioactive Glasses: Further Insights Applying Anomaly Detection. *Int. J. Mol. Sci.* 2024, *25*, 3560

**DOI:** 10.3390/ijms26020602

**Published:** 2025-01-13

**Authors:** Mauro Nascimben, Hugo Abreu, Marcello Manfredi, Giuseppe Cappellano, Annalisa Chiocchetti, Lia Rimondini

**Affiliations:** 1 Center for Translational Research on Autoimmune and Allergic Diseases, Department of Health Sciences, Università del Piemonte Orientale UPO, 28100 Novara, Italy; mauro.nascimben@uniupo.it (M.N.); hugo.abreu@uniupo.it (H.A.); giuseppe.cappellano@med.uniupo.it (G.C.); annalisa.chiocchetti@med.uniupo.it (A.C.); 2 Interdisciplinary Research Center of Autoimmune Diseases, Department of Health Sciences, Università del Piemonte Orientale UPO, 28100 Novara, Italy; 3 Biological Mass Spectrometry Laboratory, Department of Translational Medicine, Università del Piemonte Orientale UPO, 28100 Novara, Italy; marcello.manfredi@uniupo.it

In the original publication [1], there was a mistake in reporting certain row values of the “Components” column in Tables 9 and 10. The revised Table 9, with a modified caption “Nominal compositions of SBA2 and SBA3 control bioactive glasses”, and Table 10, with a modified caption “Nominal compositions of the ST control bioactive glass and ST after ion doping”, appear below. The column name “Components” is replaced by “mol.%” (mole fractions). Moreover, Table 9 and Table 10 were re-arranged horizontally.

The following correction has been made to Section 4. Materials and Methods, 4.1. Bioactive Glasses Preparation:

In the present study, silica-based bioactive glasses, either doped on their surface with silver- or copper-ions via ion exchange or by including tellurium oxide directly to the glass as a substitute for silica, were prepared following the descriptions in [93–95]. The compositions of SBA2 and SBA3 (undoped controls) are shown in Table 9.

Both SBA2 and SBA3 were prepared using a melting and quenching process. In short, all the components listed above were melted in a platinum crucible at 1450 °C for 1 h, after which the melt was cooled in a brass mold to obtain glass bars with a diameter of 1 cm, which were then annealed at 500 °C for 13 h and cut into discs of 2 mm thickness. The discs were polished with SiC abrasive papers up to 1200 grit to level the surfaces. For the ion-doping procedure, silver (Ag^+^) and copper (Cu^2+^) ions were incorporated onto the surface of SBA2 and SBA3, respectively, through the ion-exchange process consisting of submerging them in an aqueous solution of AgNO_3_ (0.03 M) or Cu(CH_3_COO)_2_ (0.001 M) for 1 h, at 37 °C.

Additionally, the composition of ST with 5% molar tellurium or without (in previous works denominated as Ste0 or a doped version of STe5, [94]) is described in Table 10.

The authors state that the scientific conclusions are unaffected. This correction was approved by the Academic Editor. The original publication has also been updated.

## Figures and Tables

**Table 9:** Nominal compositions of SBA2 and SBA3 control bioactive glasses.

mol.%	SiO_2_	Na_2_O	CaO	P_2_O_5_	B_2_O_3_	Al_2_O_3_
SBA2	48	18	30	3	0.43	0.57
SBA3	48	26	22	3	0.43	0.57

**Table 10:** Nominal compositions of the ST control bioactive glass and ST after ion doping.

mol.%	SiO_2_	Na_2_O	CaO	P_2_O_5_	TeO_2_
STe0	48.6	16.7	34.2	0.5	0
STe5	43.6	16.7	34.2	0.5	5

## References

[1] Nascimben M., Abreu H., Manfredi M., Cappellano G., Chiocchetti A., Rimondini L. (2024). Extracellular Vesicle Protein Expression in Doped Bioactive Glasses: Further Insights Applying Anomaly Detection. Int. J. Mol. Sci..

